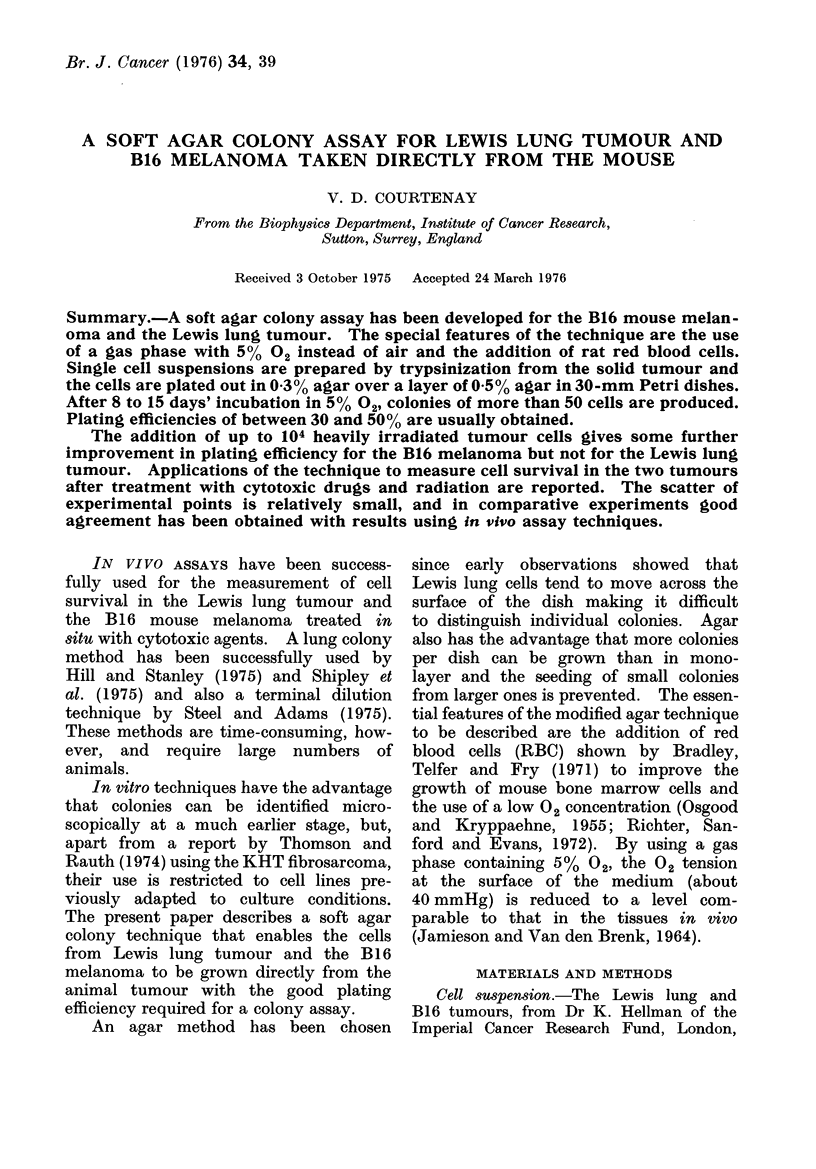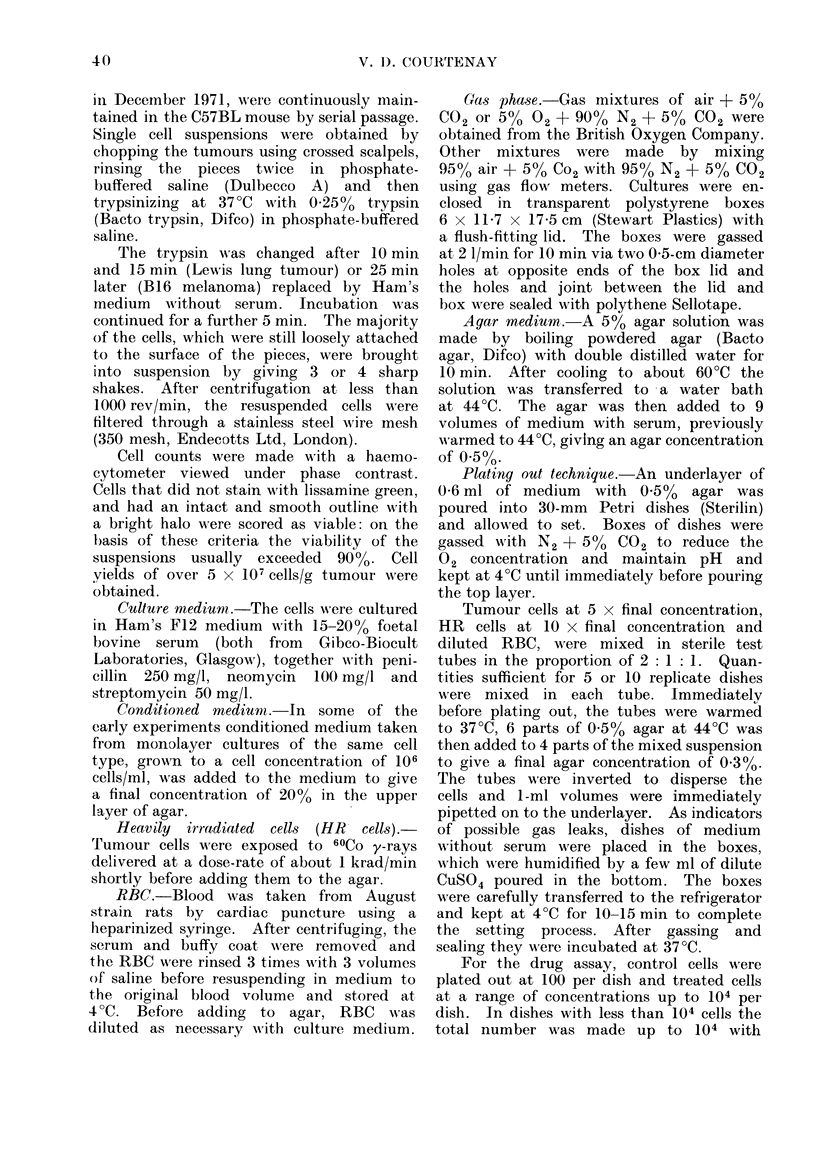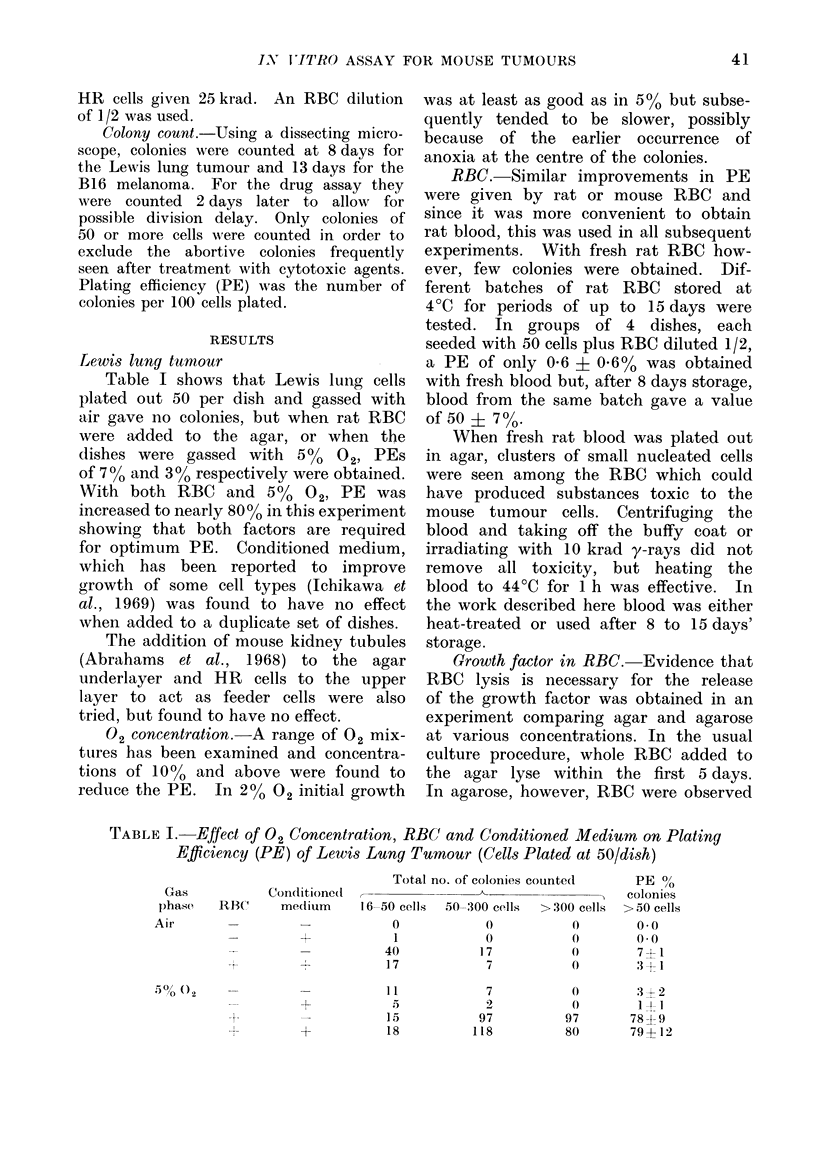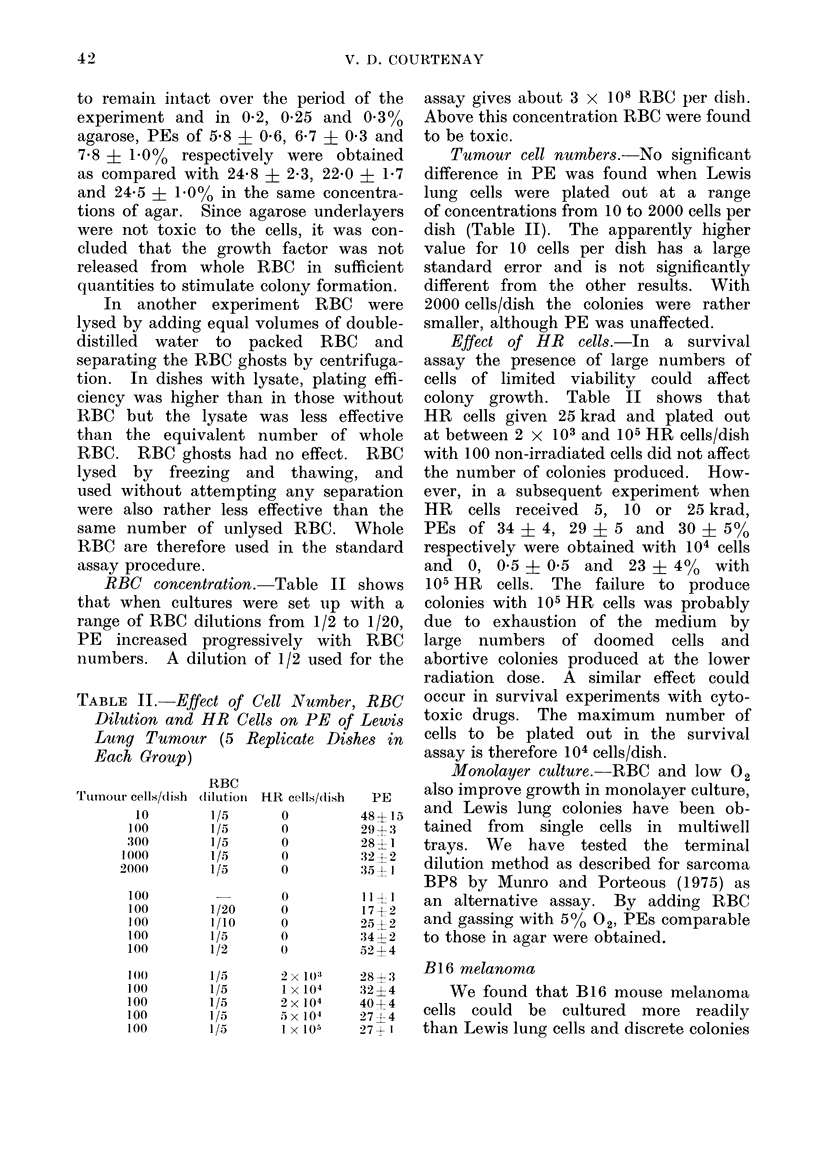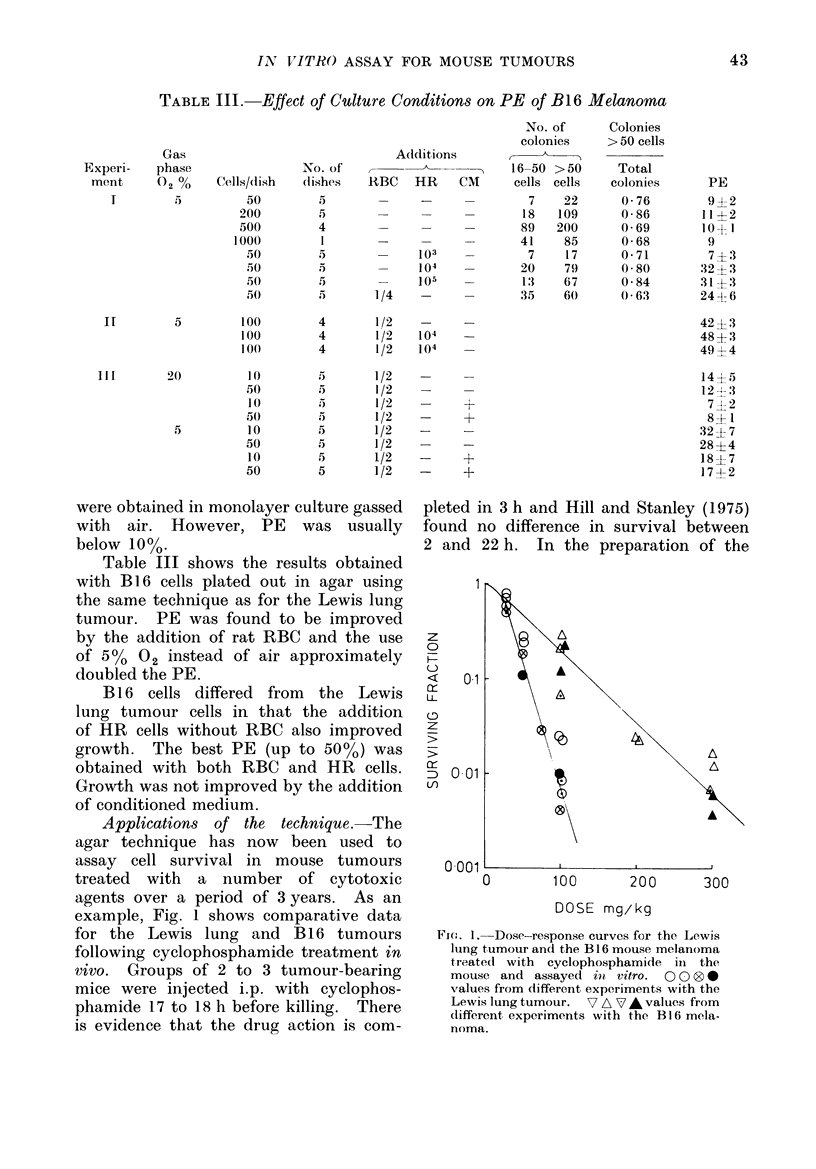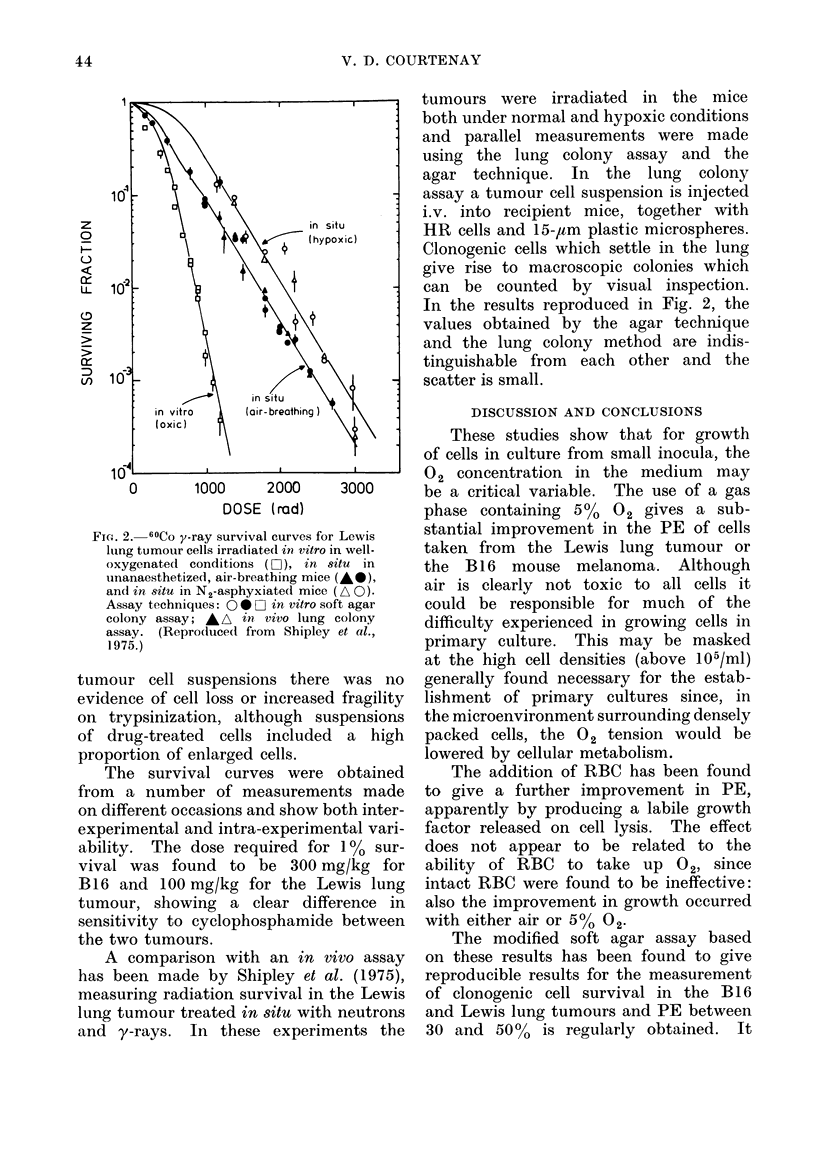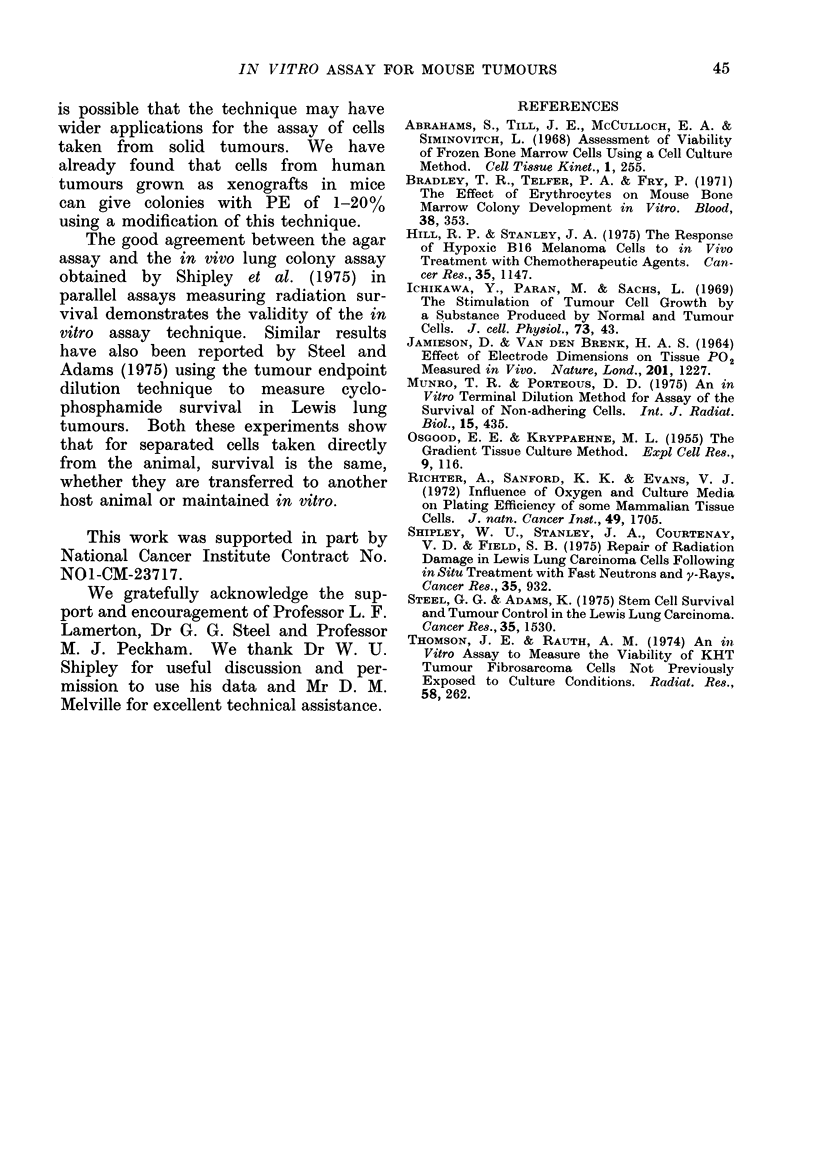# A soft agar colony assay for Lewis lung tumour and B16 melanoma taken directly from the mouse.

**DOI:** 10.1038/bjc.1976.119

**Published:** 1976-07

**Authors:** V. D. Courtenay

## Abstract

A soft agar colony assay has been developed for the B16 mouse melanoma and the Lewis lung tumour. The special features of the technique are the use of a gas phase with 5% O2 instead of air and the addition of rat red blood cells. Single cell suspensions are prepared by trypsinization from the solid tumour and the cells are plated out in 0-3% agar over a layer of 0-5% agar in 30-mm Petri dishes. After 8 to 15 days' incubation in 5% O2, colonies of more than 50 cells are produced. Plating efficiencies of between 30 and 50% are usually obtained. The addition of up to 10(4) heavily irradiated tumour cells gives some further improvement in plating efficiency for the B16 melanoma but not for the Lewis lung tumour. Applications of the technique to measure cell survival in the two tumours after treatment with cytotoxic drugs and radiation are reported. The scatter of experimental points is relatively small, and in comparative experiments good agreement has been obtained with results using in vivo assay techniques.


*Br. J. Cancer* (1976) **34**, 39

### A SOFT AGAR COLONY ASSAY FOR LEWIS LUNG TUMOUR AND B16 MELANOMA TAKEN DIRECTLY FROM THE MOUSE

V. D. COURTENAY

*From the Biophysics Department, Institute of Cancer Research, Sutton, Surrey, England*

Received 3 October 1975 Accepted 24 March 1976

**Summary.—A soft agar colony assay has been developed for the B16 mouse melanoma and the Lewis lung tumour. The special features of the technique are the use of a gas phase with 5% $O_2$ instead of air and the addition of rat red blood cells. Single cell suspensions are prepared by trypsinization from the solid tumour and the cells are plated out in 0·3% agar over a layer of 0·5% agar in 30-mm Petri dishes. After 8 to 15 days' incubation in 5% $O_2$, colonies of more than 50 cells are produced. Plating efficiencies of between 30 and 50% are usually obtained.**

**The addition of up to $10^4$ heavily irradiated tumour cells gives some further improvement in plating efficiency for the B16 melanoma but not for the Lewis lung tumour. Applications of the technique to measure cell survival in the two tumours after treatment with cytotoxic drugs and radiation are reported. The scatter of experimental points is relatively small, and in comparative experiments good agreement has been obtained with results using *in vivo* assay techniques.**

*IN VIVO* ASSAYS have been successfully used for the measurement of cell survival in the Lewis lung tumour and the B16 mouse melanoma treated *in situ* with cytotoxic agents. A lung colony method has been successfully used by Hill and Stanley (1975) and Shipley *et al.* (1975) and also a terminal dilution technique by Steel and Adams (1975). These methods are time-consuming, however, and require large numbers of animals.

*In vitro* techniques have the advantage that colonies can be identified microscopically at a much earlier stage, but, apart from a report by Thomson and Rauth (1974) using the KHT fibrosarcoma, their use is restricted to cell lines previously adapted to culture conditions. The present paper describes a soft agar colony technique that enables the cells from Lewis lung tumour and the B16 melanoma to be grown directly from the animal tumour with the good plating efficiency required for a colony assay.

An agar method has been chosen since early observations showed that Lewis lung cells tend to move across the surface of the dish making it difficult to distinguish individual colonies. Agar also has the advantage that more colonies per dish can be grown than in monolayer and the seeding of small colonies from larger ones is prevented. The essential features of the modified agar technique to be described are the addition of red blood cells (RBC) shown by Bradley, Telfer and Fry (1971) to improve the growth of mouse bone marrow cells and the use of a low $O_2$ concentration (Osgood and Kryppaehne, 1955; Richter, Sanford and Evans, 1972). By using a gas phase containing 5% $O_2$, the $O_2$ tension at the surface of the medium (about 40 mmHg) is reduced to a level comparable to that in the tissues *in vivo* (Jamieson and Van den Brenk, 1964).

### MATERIALS AND METHODS

*Cell suspension.*—The Lewis lung and B16 tumours, from Dr K. Hellman of the Imperial Cancer Research Fund, London, in December 1971, were continuously maintained in the C57BL mouse by serial passage. Single cell suspensions were obtained by chopping the tumours using crossed scalpels, rinsing the pieces twice in phosphate-buffered saline (Dulbecco A) and then trypsinizing at 37°C with 0·25% trypsin (Bacto trypsin, Difco) in phosphate-buffered saline.

The trypsin was changed after 10 min and 15 min (Lewis lung tumour) or 25 min later (B16 melanoma) replaced by Ham's medium without serum. Incubation was continued for a further 5 min. The majority of the cells, which were still loosely attached to the surface of the pieces, were brought into suspension by giving 3 or 4 sharp shakes. After centrifugation at less than 1000 rev/min, the resuspended cells were filtered through a stainless steel wire mesh (350 mesh, Endecotts Ltd, London).

Cell counts were made with a haemocytometer viewed under phase contrast. Cells that did not stain with lissamine green, and had an intact and smooth outline with a bright halo were scored as viable: on the basis of these criteria the viability of the suspensions usually exceeded 90%. Cell yields of over $5 \times 10^7$ cells/g tumour were obtained.

*Culture medium.*—The cells were cultured in Ham's F12 medium with 15–20% foetal bovine serum (both from Gibco-Biocult Laboratories, Glasgow), together with penicillin 250 mg/l, neomycin 100 mg/l and streptomycin 50 mg/l.

*Conditioned medium.*—In some of the early experiments conditioned medium taken from monolayer cultures of the same cell type, grown to a cell concentration of $10^6$ cells/ml, was added to the medium to give a final concentration of 20% in the upper layer of agar.

*Heavily irradiated cells (HR cells).*—Tumour cells were exposed to $^{60}$Co $\gamma$-rays delivered at a dose-rate of about 1 krad/min shortly before adding them to the agar.

*RBC.*—Blood was taken from August strain rats by cardiac puncture using a heparinized syringe. After centrifuging, the serum and buffy coat were removed and the RBC were rinsed 3 times with 3 volumes of saline before resuspending in medium to the original blood volume and stored at 4°C. Before adding to agar, RBC was diluted as necessary with culture medium.

*Gas phase.*—Gas mixtures of air + 5% $CO_2$ or 5% $O_2$ + 90% $N_2$ + 5% $CO_2$ were obtained from the British Oxygen Company. Other mixtures were made by mixing 95% air + 5% $Co_2$ with 95% $N_2$ + 5% $CO_2$ using gas flow meters. Cultures were enclosed in transparent polystyrene boxes $6 \times 11{\cdot}7 \times 17{\cdot}5$ cm (Stewart Plastics) with a flush-fitting lid. The boxes were gassed at 2 l/min for 10 min via two 0·5-cm diameter holes at opposite ends of the box lid and the holes and joint between the lid and box were sealed with polythene Sellotape.

*Agar medium.*—A 5% agar solution was made by boiling powdered agar (Bacto agar, Difco) with double distilled water for 10 min. After cooling to about 60°C the solution was transferred to a water bath at 44°C. The agar was then added to 9 volumes of medium with serum, previously warmed to 44°C, givlng an agar concentration of 0·5%.

*Plating out technique.*—An underlayer of 0·6 ml of medium with 0·5% agar was poured into 30-mm Petri dishes (Sterilin) and allowed to set. Boxes of dishes were gassed with $N_2$ + 5% $CO_2$ to reduce the $O_2$ concentration and maintain pH and kept at 4°C until immediately before pouring the top layer.

Tumour cells at 5 × final concentration, HR cells at 10 × final concentration and diluted RBC, were mixed in sterile test tubes in the proportion of 2 : 1 : 1. Quantities sufficient for 5 or 10 replicate dishes were mixed in each tube. Immediately before plating out, the tubes were warmed to 37°C, 6 parts of 0·5% agar at 44°C was then added to 4 parts of the mixed suspension to give a final agar concentration of 0·3%. The tubes were inverted to disperse the cells and 1-ml volumes were immediately pipetted on to the underlayer. As indicators of possible gas leaks, dishes of medium without serum were placed in the boxes, which were humidified by a few ml of dilute $CuSO_4$ poured in the bottom. The boxes were carefully transferred to the refrigerator and kept at 4°C for 10–15 min to complete the setting process. After gassing and sealing they were incubated at 37°C.

For the drug assay, control cells were plated out at 100 per dish and treated cells at a range of concentrations up to $10^4$ per dish. In dishes with less than $10^4$ cells the total number was made up to $10^4$ with

HR cells given 25 krad. An RBC dilution of 1/2 was used.

*Colony count.*—Using a dissecting microscope, colonies were counted at 8 days for the Lewis lung tumour and 13 days for the B16 melanoma. For the drug assay they were counted 2 days later to allow for possible division delay. Only colonies of 50 or more cells were counted in order to exclude the abortive colonies frequently seen after treatment with cytotoxic agents. Plating efficiency (PE) was the number of colonies per 100 cells plated.

### RESULTS

### *Lewis lung tumour*

Table I shows that Lewis lung cells plated out 50 per dish and gassed with air gave no colonies, but when rat RBC were added to the agar, or when the dishes were gassed with 5% $O_2$, PEs of 7% and 3% respectively were obtained. With both RBC and 5% $O_2$, PE was increased to nearly 80% in this experiment showing that both factors are required for optimum PE. Conditioned medium, which has been reported to improve growth of some cell types (Ichikawa *et al.*, 1969) was found to have no effect when added to a duplicate set of dishes.

The addition of mouse kidney tubules (Abrahams *et al.*, 1968) to the agar underlayer and HR cells to the upper layer to act as feeder cells were also tried, but found to have no effect.

*$O_2$ concentration.*—A range of $O_2$ mixtures has been examined and concentrations of 10% and above were found to reduce the PE. In 2% $O_2$ initial growth was at least as good as in 5% but subsequently tended to be slower, possibly because of the earlier occurrence of anoxia at the centre of the colonies.

*RBC.*—Similar improvements in PE were given by rat or mouse RBC and since it was more convenient to obtain rat blood, this was used in all subsequent experiments. With fresh rat RBC however, few colonies were obtained. Different batches of rat RBC stored at 4°C for periods of up to 15 days were tested. In groups of 4 dishes, each seeded with 50 cells plus RBC diluted 1/2, a PE of only 0·6 ± 0·6% was obtained with fresh blood but, after 8 days storage, blood from the same batch gave a value of 50 ± 7%.

When fresh rat blood was plated out in agar, clusters of small nucleated cells were seen among the RBC which could have produced substances toxic to the mouse tumour cells. Centrifuging the blood and taking off the buffy coat or irradiating with 10 krad $\gamma$-rays did not remove all toxicity, but heating the blood to 44°C for 1 h was effective. In the work described here blood was either heat-treated or used after 8 to 15 days' storage.

*Growth factor in RBC.*—Evidence that RBC lysis is necessary for the release of the growth factor was obtained in an experiment comparing agar and agarose at various concentrations. In the usual culture procedure, whole RBC added to the agar lyse within the first 5 days. In agarose, however, RBC were observed

TABLE I.—*Effect of $O_2$ Concentration, RBC and Conditioned Medium on Plating Efficiency (PE) of Lewis Lung Tumour (Cells Plated at 50/dish)*

| Gas phase | RBC | Conditioned medium | Total no. of colonies counted: 16–50 cells | 50–300 cells | >300 cells | PE % colonies >50 cells |
|---|---|---|---|---|---|---|
| Air | − | − | 0 | 0 | 0 | 0·0 |
| | − | + | 1 | 0 | 0 | 0·0 |
| | + | − | 40 | 17 | 0 | 7±1 |
| | + | + | 17 | 7 | 0 | 3±1 |
| 5% $O_2$ | − | − | 11 | 7 | 0 | 3±2 |
| | − | + | 5 | 2 | 0 | 1±1 |
| | + | − | 15 | 97 | 97 | 78±9 |
| | + | + | 18 | 118 | 80 | 79±12 |

to remain intact over the period of the experiment and in 0·2, 0·25 and 0·3% agarose, PEs of 5·8 ± 0·6, 6·7 ± 0·3 and 7·8 ± 1·0% respectively were obtained as compared with 24·8 ± 2·3, 22·0 ± 1·7 and 24·5 ± 1·0% in the same concentrations of agar. Since agarose underlayers were not toxic to the cells, it was concluded that the growth factor was not released from whole RBC in sufficient quantities to stimulate colony formation.

In another experiment RBC were lysed by adding equal volumes of double-distilled water to packed RBC and separating the RBC ghosts by centrifugation. In dishes with lysate, plating efficiency was higher than in those without RBC but the lysate was less effective than the equivalent number of whole RBC. RBC ghosts had no effect. RBC lysed by freezing and thawing, and used without attempting any separation were also rather less effective than the same number of unlysed RBC. Whole RBC are therefore used in the standard assay procedure.

*RBC concentration.*—Table II shows that when cultures were set up with a range of RBC dilutions from 1/2 to 1/20, PE increased progressively with RBC numbers. A dilution of 1/2 used for the assay gives about $3 \times 10^8$ RBC per dish. Above this concentration RBC were found to be toxic.

TABLE II.—*Effect of Cell Number, RBC Dilution and HR Cells on PE of Lewis Lung Tumour* (5 *Replicate Dishes in Each Group*)

| Tumour cells/dish | RBC dilution | HR cells/dish | PE |
|---|---|---|---|
| 10 | 1/5 | 0 | 48 ± 15 |
| 100 | 1/5 | 0 | 29 ± 3 |
| 300 | 1/5 | 0 | 28 ± 1 |
| 1000 | 1/5 | 0 | 32 ± 2 |
| 2000 | 1/5 | 0 | 35 ± 1 |
| 100 | — | 0 | 11 ± 1 |
| 100 | 1/20 | 0 | 17 ± 2 |
| 100 | 1/10 | 0 | 25 ± 2 |
| 100 | 1/5 | 0 | 34 ± 2 |
| 100 | 1/2 | 0 | 52 ± 4 |
| 100 | 1/5 | $2 \times 10^3$ | 28 ± 3 |
| 100 | 1/5 | $1 \times 10^4$ | 32 ± 4 |
| 100 | 1/5 | $2 \times 10^4$ | 40 ± 4 |
| 100 | 1/5 | $5 \times 10^4$ | 27 ± 4 |
| 100 | 1/5 | $1 \times 10^5$ | 27 ± 1 |

*Tumour cell numbers.*—No significant difference in PE was found when Lewis lung cells were plated out at a range of concentrations from 10 to 2000 cells per dish (Table II). The apparently higher value for 10 cells per dish has a large standard error and is not significantly different from the other results. With 2000 cells/dish the colonies were rather smaller, although PE was unaffected.

*Effect of HR cells.*—In a survival assay the presence of large numbers of cells of limited viability could affect colony growth. Table II shows that HR cells given 25 krad and plated out at between $2 \times 10^3$ and $10^5$ HR cells/dish with 100 non-irradiated cells did not affect the number of colonies produced. However, in a subsequent experiment when HR cells received 5, 10 or 25 krad, PEs of 34 ± 4, 29 ± 5 and 30 ± 5% respectively were obtained with $10^4$ cells and 0, 0·5 ± 0·5 and 23 ± 4% with $10^5$ HR cells. The failure to produce colonies with $10^5$ HR cells was probably due to exhaustion of the medium by large numbers of doomed cells and abortive colonies produced at the lower radiation dose. A similar effect could occur in survival experiments with cytotoxic drugs. The maximum number of cells to be plated out in the survival assay is therefore $10^4$ cells/dish.

*Monolayer culture.*—RBC and low $O_2$ also improve growth in monolayer culture, and Lewis lung colonies have been obtained from single cells in multiwell trays. We have tested the terminal dilution method as described for sarcoma BP8 by Munro and Porteous (1975) as an alternative assay. By adding RBC and gassing with 5% $O_2$, PEs comparable to those in agar were obtained.

*B16 melanoma*

We found that B16 mouse melanoma cells could be cultured more readily than Lewis lung cells and discrete colonies

TABLE III.—*Effect of Culture Conditions on PE of B*16 *Melanoma*

| Experiment | Gas phase $O_2$ % | Cells/dish | No. of dishes | Additions RBC | Additions HR | Additions CM | No. of colonies 16–50 cells | No. of colonies >50 cells | Colonies >50 cells / Total colonies | PE |
|---|---|---|---|---|---|---|---|---|---|---|
| I | 5 | 50 | 5 | – | – | – | 7 | 22 | 0·76 | 9±2 |
| | | 200 | 5 | – | – | – | 18 | 109 | 0·86 | 11±2 |
| | | 500 | 4 | – | – | – | 89 | 200 | 0·69 | 10±1 |
| | | 1000 | 1 | – | – | – | 41 | 85 | 0·68 | 9 |
| | | 50 | 5 | – | $10^3$ | – | 7 | 17 | 0·71 | 7±3 |
| | | 50 | 5 | – | $10^4$ | – | 20 | 79 | 0·80 | 32±3 |
| | | 50 | 5 | – | $10^5$ | – | 13 | 67 | 0·84 | 31±3 |
| | | 50 | 5 | 1/4 | – | – | 35 | 60 | 0·63 | 24±6 |
| II | 5 | 100 | 4 | 1/2 | – | – | | | | 42±3 |
| | | 100 | 4 | 1/2 | $10^4$ | – | | | | 48±3 |
| | | 100 | 4 | 1/2 | $10^4$ | – | | | | 49±4 |
| III | 20 | 10 | 5 | 1/2 | – | – | | | | 14±5 |
| | | 50 | 5 | 1/2 | – | – | | | | 12±3 |
| | | 10 | 5 | 1/2 | – | + | | | | 7±2 |
| | | 50 | 5 | 1/2 | – | + | | | | 8±1 |
| | 5 | 10 | 5 | 1/2 | – | – | | | | 32±7 |
| | | 50 | 5 | 1/2 | – | – | | | | 28±4 |
| | | 10 | 5 | 1/2 | – | + | | | | 18±7 |
| | | 50 | 5 | 1/2 | – | + | | | | 17±2 |

were obtained in monolayer culture gassed with air. However, PE was usually below 10%.

Table III shows the results obtained with B16 cells plated out in agar using the same technique as for the Lewis lung tumour. PE was found to be improved by the addition of rat RBC and the use of 5% $O_2$ instead of air approximately doubled the PE.

B16 cells differed from the Lewis lung tumour cells in that the addition of HR cells without RBC also improved growth. The best PE (up to 50%) was obtained with both RBC and HR cells. Growth was not improved by the addition of conditioned medium.

*Applications of the technique.*—The agar technique has now been used to assay cell survival in mouse tumours treated with a number of cytotoxic agents over a period of 3 years. As an example, Fig. 1 shows comparative data for the Lewis lung and B16 tumours following cyclophosphamide treatment *in vivo*. Groups of 2 to 3 tumour-bearing mice were injected i.p. with cyclophosphamide 17 to 18 h before killing. There is evidence that the drug action is completed in 3 h and Hill and Stanley (1975) found no difference in survival between 2 and 22 h. In the preparation of the

FIG. 1.—Dose–response curves for the Lewis lung tumour and the B16 mouse melanoma treated with cyclophosphamide in the mouse and assayed *in vitro*. ○ ○ ⊗ ● values from different experiments with the Lewis lung tumour. ▽ △ ▽ ▲ values from different experiments with the B16 melanoma.

FIG. 2.—$^{60}Co$ $\gamma$-ray survival curves for Lewis lung tumour cells irradiated *in vitro* in well-oxygenated conditions (□), *in situ* in unanaesthetized, air-breathing mice (▲●), and *in situ* in $N_2$-asphyxiated mice (△○). Assay techniques: ○●□ *in vitro* soft agar colony assay; ▲△ *in vivo* lung colony assay. (Reproduced from Shipley *et al.*, 1975.)

tumour cell suspensions there was no evidence of cell loss or increased fragility on trypsinization, although suspensions of drug-treated cells included a high proportion of enlarged cells.

The survival curves were obtained from a number of measurements made on different occasions and show both inter-experimental and intra-experimental variability. The dose required for 1% survival was found to be 300 mg/kg for B16 and 100 mg/kg for the Lewis lung tumour, showing a clear difference in sensitivity to cyclophosphamide between the two tumours.

A comparison with an *in vivo* assay has been made by Shipley *et al.* (1975), measuring radiation survival in the Lewis lung tumour treated *in situ* with neutrons and $\gamma$-rays. In these experiments the tumours were irradiated in the mice both under normal and hypoxic conditions and parallel measurements were made using the lung colony assay and the agar technique. In the lung colony assay a tumour cell suspension is injected i.v. into recipient mice, together with HR cells and 15-$\mu$m plastic microspheres. Clonogenic cells which settle in the lung give rise to macroscopic colonies which can be counted by visual inspection. In the results reproduced in Fig. 2, the values obtained by the agar technique and the lung colony method are indistinguishable from each other and the scatter is small.

### DISCUSSION AND CONCLUSIONS

These studies show that for growth of cells in culture from small inocula, the $O_2$ concentration in the medium may be a critical variable. The use of a gas phase containing 5% $O_2$ gives a substantial improvement in the PE of cells taken from the Lewis lung tumour or the B16 mouse melanoma. Although air is clearly not toxic to all cells it could be responsible for much of the difficulty experienced in growing cells in primary culture. This may be masked at the high cell densities (above $10^5$/ml) generally found necessary for the establishment of primary cultures since, in the microenvironment surrounding densely packed cells, the $O_2$ tension would be lowered by cellular metabolism.

The addition of RBC has been found to give a further improvement in PE, apparently by producing a labile growth factor released on cell lysis. The effect does not appear to be related to the ability of RBC to take up $O_2$, since intact RBC were found to be ineffective: also the improvement in growth occurred with either air or 5% $O_2$.

The modified soft agar assay based on these results has been found to give reproducible results for the measurement of clonogenic cell survival in the B16 and Lewis lung tumours and PE between 30 and 50% is regularly obtained. It is possible that the technique may have wider applications for the assay of cells taken from solid tumours. We have already found that cells from human tumours grown as xenografts in mice can give colonies with PE of 1–20% using a modification of this technique.

The good agreement between the agar assay and the *in vivo* lung colony assay obtained by Shipley *et al.* (1975) in parallel assays measuring radiation survival demonstrates the validity of the *in vitro* assay technique. Similar results have also been reported by Steel and Adams (1975) using the tumour endpoint dilution technique to measure cyclophosphamide survival in Lewis lung tumours. Both these experiments show that for separated cells taken directly from the animal, survival is the same, whether they are transferred to another host animal or maintained *in vitro*.

This work was supported in part by National Cancer Institute Contract No. NO1-CM-23717.

We gratefully acknowledge the support and encouragement of Professor L. F. Lamerton, Dr G. G. Steel and Professor M. J. Peckham. We thank Dr W. U. Shipley for useful discussion and permission to use his data and Mr D. M. Melville for excellent technical assistance.

REFERENCES

Abrahams, S., Till, J. E., McCulloch, E. A. & Siminovitch, L. (1968) Assessment of Viability of Frozen Bone Marrow Cells Using a Cell Culture Method. *Cell Tissue Kinet.*, **1**, 255.

Bradley, T. R., Telfer, P. A. & Fry, P. (1971) The Effect of Erythrocytes on Mouse Bone Marrow Colony Development *in Vitro*. *Blood*, **38**, 353.

Hill, R. P. & Stanley, J. A. (1975) The Response of Hypoxic B16 Melanoma Cells to *in Vivo* Treatment with Chemotherapeutic Agents. *Cancer Res.*, **35**, 1147.

Ichikawa, Y., Paran, M. & Sachs, L. (1969) The Stimulation of Tumour Cell Growth by a Substance Produced by Normal and Tumour Cells. *J. cell. Physiol.*, **73**, 43.

Jamieson, D. & Van den Brenk, H. A. S. (1964) Effect of Electrode Dimensions on Tissue $PO_2$ Measured *in Vivo*. *Nature, Lond.*, **201**, 1227.

Munro, T. R. & Porteous, D. D. (1975) An *in Vitro* Terminal Dilution Method for Assay of the Survival of Non-adhering Cells. *Int. J. Radiat. Biol.*, **15**, 435.

Osgood, E. E. & Kryppaehne, M. L. (1955) The Gradient Tissue Culture Method. *Expl Cell Res.*, **9**, 116.

Richter, A., Sanford, K. K. & Evans, V. J. (1972) Influence of Oxygen and Culture Media on Plating Efficiency of some Mammalian Tissue Cells. *J. natn. Cancer Inst.*, **49**, 1705.

Shipley, W. U., Stanley, J. A., Courtenay, V. D. & Field, S. B. (1975) Repair of Radiation Damage in Lewis Lung Carcinoma Cells Following *in Situ* Treatment with Fast Neutrons and $\gamma$-Rays. *Cancer Res.*, **35**, 932.

Steel, G. G. & Adams, K. (1975) Stem Cell Survival and Tumour Control in the Lewis Lung Carcinoma. *Cancer Res.*, **35**, 1530.

Thomson, J. E. & Rauth, A. M. (1974) An *in Vitro* Assay to Measure the Viability of KHT Tumour Fibrosarcoma Cells Not Previously Exposed to Culture Conditions. *Radiat. Res.*, **58**, 262.